# Development of a blood-based gene expression algorithm for assessment of obstructive coronary artery disease in non-diabetic patients

**DOI:** 10.1186/1755-8794-4-26

**Published:** 2011-03-28

**Authors:** Michael R Elashoff, James A Wingrove, Philip Beineke, Susan E Daniels, Whittemore G Tingley, Steven Rosenberg, Szilard Voros, William E Kraus, Geoffrey S Ginsburg, Robert S Schwartz, Stephen G Ellis, Naheem Tahirkheli, Ron Waksman, John McPherson, Alexandra J Lansky, Eric J Topol

**Affiliations:** 1 CardioDx, Inc., 2500 Faber Place, Palo Alto, CA 94602 USA; 2 Division of Cardiology, University of California, San Francisco, CA 94143 USA; 3 Fuqua Heart Center, Piedmont Heart Institute, Atlanta, GA 30309 USA; 4 Department of Cardiology and Center for Genomic Medicine, Duke University School of Medicine, Durham, NC 27710 USA; 5 Minneapolis Heart Institute and Foundation, Minneapolis, MN 55407 USA; 6 Department of Cardiovascular Medicine, Cleveland Clinic Foundation, Cleveland, OH 44195 USA; 7 Oklahoma Cardiovascular Research Group, Oklahoma City, OK 73109 USA; 8 Cardiovascular Research Institute, Medstar Research Institute, Washington, DC 20010 USA; 9 Department of Medicine, Vanderbilt Heart and Vascular Institute, Nashville, TN 37232 USA; 10 Department of Medicine, Yale University Medical Center, New Haven, CT 06520 USA; 11 Scripps Translational Science Institute, La Jolla, CA 92037 USA

**Keywords:** Atherosclerosis, gene expression, whole blood classifier

## Abstract

**Background:**

Alterations in gene expression in peripheral blood cells have been shown to be sensitive to the presence and extent of coronary artery disease (CAD). A non-invasive blood test that could reliably assess obstructive CAD likelihood would have diagnostic utility.

**Results:**

Microarray analysis of RNA samples from a 195 patient Duke CATHGEN registry case:control cohort yielded 2,438 genes with significant CAD association (p < 0.05), and identified the clinical/demographic factors with the largest effects on gene expression as age, sex, and diabetic status. RT-PCR analysis of 88 CAD classifier genes confirmed that diabetic status was the largest clinical factor affecting CAD associated gene expression changes. A second microarray cohort analysis limited to non-diabetics from the multi-center PREDICT study (198 patients; 99 case: control pairs matched for age and sex) evaluated gene expression, clinical, and cell population predictors of CAD and yielded 5,935 CAD genes (p < 0.05) with an intersection of 655 genes with the CATHGEN results. Biological pathway (gene ontology and literature) and statistical analyses (hierarchical clustering and logistic regression) were used in combination to select 113 genes for RT-PCR analysis including CAD classifiers, cell-type specific markers, and normalization genes.

RT-PCR analysis of these 113 genes in a PREDICT cohort of 640 non-diabetic subject samples was used for algorithm development. Gene expression correlations identified clusters of CAD classifier genes which were reduced to meta-genes using LASSO. The final classifier for assessment of obstructive CAD was derived by Ridge Regression and contained sex-specific age functions and 6 meta-gene terms, comprising 23 genes. This algorithm showed a cross-validated estimated AUC = 0.77 (95% CI 0.73-0.81) in ROC analysis.

**Conclusions:**

We have developed a whole blood classifier based on gene expression, age and sex for the assessment of obstructive CAD in non-diabetic patients from a combination of microarray and RT-PCR data derived from studies of patients clinically indicated for invasive angiography.

**Clinical trial registration information:**

PREDICT, Personalized Risk Evaluation and Diagnosis in the Coronary Tree, http://www.clinicaltrials.gov, NCT00500617

## Background

The promise of genomics to improve diagnosis and prognosis of significant diseases is dependent on a number of factors including appropriate use of technology, identification of clinical issues of significant unmet need, and the rigorous statistical derivation and testing of genomic classifiers[1]. Multigene expression classifiers have been developed and have become incorporated into clinical guidelines in both breast cancer recurrence prognosis and heart transplant rejection monitoring[2,3]. A guideline for the metrics such classifiers should meet, including independent validation, and adding to current clinical factor algorithms has been described [4] and it has been suggested that peripheral blood cell gene expression may reflect pathological conditions in a variety of cardiovascular disease states[5]. In this work we describe the development of a validated whole blood based classifier for the assessment of obstructive CAD[6].

Mortality and morbidity from CAD and myocardial infarction (MI) are a major global health burden. Major determinants of current CAD likelihood are sex, age, and chest-pain type [7,8]. Other risk factors such as diabetes, smoking, dyslipidemia, hypertension and family history have been associated with future cardiovascular event risk[9]. In addition, atherosclerosis has a systemic inflammatory component including activation and migration of immune cells into the vessel wall[10,11]. Prior work has shown that quantitative measurements of circulating blood cell gene expression reflect the extent of CAD[12,13]. These observations likely reflect both changes in cell type distributions, which have prognostic value for cardiovascular events [14] and gene expression changes within a specific cell type or lineage.

The "gold standard" for detecting CAD is invasive coronary angiography; however, this is costly, and can pose risk to the patient. Prior to angiography, non-invasive diagnostic modalities such as myocardial perfusion imaging (MPI) and CT-angiography may be used, however these only add moderately to obstructive CAD identification[15]. We describe herein the development of an algorithm for the assessment of obstructive CAD using peripheral blood gene expression, age, and sex, which was subsequently validated in an independent cohort[6].

## Methods

### Patient selection and clinical methods

All patients were clinically referred for angiography and angiograms were performed based on local, institutional protocols. The first microarray cohort of 198 subjects (88 cases and 110 controls) was derived from the Duke University CATHGEN registry, a retrospective blood repository, enrolled between August 2004 and November, 2005 [16]. For CATHGEN patients, clinical angiographic interpretation defined cases as ≥75% maximum stenosis in one major vessel or ≥50% in two vessels and controls as <25% stenosis in all major vessels. Clinical inclusion and exclusion criteria were described previously and included both diabetic and non-diabetic patients [13]. All CATHGEN patients gave written informed consent and the study protocol was approved by the Duke University IRB.

The second microarray cohort of 210 subjects (105 case: control pairs, matched for age and sex) and the RT-PCR algorithm development cohort (210 cases and 430 controls) were part of PREDICT, a multi-center US study of patients referred for coronary angiography (http://www.clinicaltrials.gov, NCT00500617). For PREDICT patients, core laboratory QCA reads (Cardiovascular Research Foundation New York) were used for case: control classification. Cases had ≥50% stenosis in at least one major coronary vessel and controls <50% stenosis in all major vessels.

Subjects from PREDICT were eligible if they had a history of chest pain, suspected anginal-equivalent symptoms, or a high risk of CAD with no known prior MI, revascularization, or CAD. Detailed inclusion/exclusion criteria have been described [6]. Diabetic status was defined by clinical identification, blood glucose (non-fasting ≥200 or fasting ≥126), rorhemoglobin A1c, (≥6.5), or diabetic medication prescription. Complete blood counts with differentials were obtained for all patients. PREDICT patients gave written informed consent, and the study protocol was approved by the Institutional Review Boards.

### Blood collection, RNA purification and RT-PCR

Whole blood samples were collected in PAXgene^® ^tubes prior to coronary angiography, according to the manufacturer's instructions, then frozen at -20°C. For the CATHGEN samples RNA was purified as described (PreAnalytix, Franklin Lakes, NJ), followed by quantitative analysis (Ribogreen, Molecular Probes, Eugene, OR). For the PREDICT samples an automated method using the Agencourt RNAdvance system was employed.

### Correlation between gene expression and cell type distributions

Correlations with complete blood counts and database gene expression analysis (SymAtlas, http://biogps.gnf.org) were used to identify highly cell-type selective genes. In addition, whole blood cell fractionation by density centrifugation or through positive antibody selection followed by RT-PCR was performed on specific cell fractions (see Additional file 1).

### Statistical methods

All statistical methods were performed using the R software package.

### Microarray methods

Microarray samples were labeled and hybridized to 41K Human Whole Genome Arrays (Agilent, PN #G4112A) using the manufacturer's protocol. For PREDICT microarrays all matched pairs were labeled and hybridized together to minimize microarray batch effects. Microarray data sets have been deposited in GEO (GSE 20686).

### Normalization

Agilent processed signal values for array normalization were scaled to a trimmed mean of 100 and then log2 transformed. Standard array QC metrics (percent present, pair-wise correlation, and signal intensity) were used for quality assessment, resulting in 3 of 198 CATHGEN and 12 of 210 PREDICT samples being excluded.

### Array analysis

For the CATHGEN array, logistic regression (unadjusted and sex/age adjusted) was used to assess gene expression association with case: control status. For the PREDICT array, given the paired design, conditional logistic regression was used. False discovery rates were used to account for multiple comparisons. BINGO was used to assess enrichment of gene ontology terms in the set of 655 genes[17]. A hyper-geometric test was used to identify overrepresented terms; results were corrected for multiple testing using Benjamini & Hochberg False Discovery Rate (FDR) correction.

### Gene selection

Genes for RT-PCR were selected based on statistical significance, gene ontology pathway analysis, and literature support. Hierarchical clustering based on gene: gene correlations ensured that RT-PCR genes represented multiple clusters. Normalization genes were selected based on low variance, moderate to high expression, and no significant association with case: control status, sex, age, or cell counts. Cell-type genes were selected based on known literature or correlation to known cell-type specific markers.

### PCR methods

Amplicon design, cDNA synthesis, and RT-PCR were performed as previously described [6,13]. All PCR reactions were run in triplicate and median values used for analysis. Clinical/demographic factors were assessed for CAD association using univariate and multivariate logistic regression. Gene expression association with CAD and other clinical/demographic factors was assessed by robust logistic regression (unadjusted and sex/age adjusted) [13].

### Algorithm development and validation

Hierarchical clustering was used to group genes using a correlation cutoff. Clusters were reduced to meta-genes[18] and normalization genes based on correlation structure, known biology, and cell count correlation. In general, a meta-gene was a set of 1-4 genes from a specific cluster, chosen to best represent the cluster center using a parsimonious number of genes. Genes within meta-genes were equally weighted with one exception (Additional File 1). For meta-gene pairs with high correlation and opposite disease regulation, ratio terms (differences on the log scale) were defined. Meta-genes independently associated with outcome were selected by the LASSO method, with sex by meta-gene interactions allowed during variable selection [19].

The final algorithm was fit using Ridge regression [20], where the outcome variable was case: control status and the predictors the LASSO-selected meta-genes and sex-specific age terms. Sex was a binary predictor, and age a linear predictor with separate slopes for men, women >60, and women <60 (the slope for women age < 60 was estimated to be approximately 0 and thus was set to 0 in the final algorithm). The LASSO was fit using the glmnet package in R and ridge regression was fit using the Design package in R; in both cases the shrinkage parameter lambda was estimated using 10-fold cross validation. Model performance was estimated using leave-one-out cross-validation.

## Results

**Figure 1:**
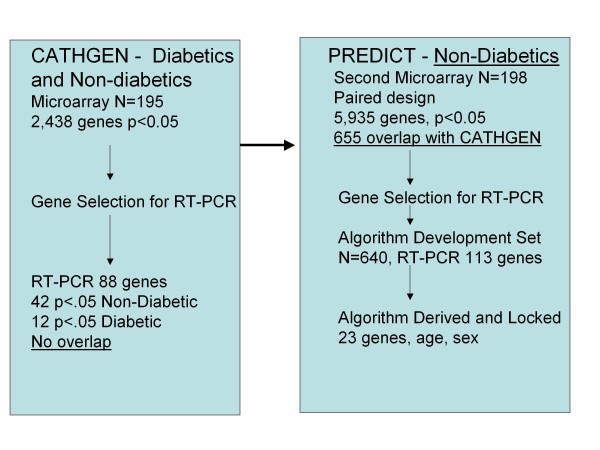
**Gene discovery and algorithm development patient and logic flow schematic**. Initial gene discovery (CATHGEN repository) included both diabetic and non-diabetic patients. Gene discovery from PREDICT involved non-diabetic patients in a paired microarray analysis, that yielded 655 significant genes in common with those from the CATHGEN arrays. For RT-PCR 113 genes were selected and tested on 640 PREDICT patient samples, from which the final algorithm was derived and locked.

A schematic of the patient, gene, and logic flow for gene discovery and algorithm development is shown in Figure 1. Baseline demographic characteristics of the CATHGEN registry and PREDICT study microarray patient cohorts are summarized in Table 1. Significant clinical and demographic factors for obstructive CAD were age, male sex, systolic blood pressure, and dyslipidemia; increased neutrophil count and decreased lymphocyte count trended toward significance. In all cases whole blood samples were obtained in PAXgene^® ^tubes and microarray analysis performed using the Agilent 41K platform.

**Table 1:** CATHGEN and PREDICT microarray cohort clinical and demographic characteristics

	CATHGEN Microarray Cohort			PREDICT Paired Microarray Cohort		
	Controls	Cases		Controls	Cases	
Variable	(N = 108)	(N = 87)	p.value	(N = 99)	(N = 99)	p.value
Sex (%Male)	55 (50.9%)	58 (66.7%)	0.039	75(75.8%)	75 (75.8%)	0.868
Age (yrs)	55 ± 11	63 ± 10	<.001	55 ± 12	62 ± 11	<.001
Caucasian	56 (51.9%)	60 (69%)	0.023	85(85.9%)	92 (92.9%)	0.166
BMI	32 ± 7	30 ± 6	0.098	30 ± 7	30 ± 6	0.722
Current Smoker	41 (38%)	45 (51.7%)	0.075	14(14.1%)	25 (25.3%)	0.074
Systolic BP	144 ± 22	153 ± 25	0.007	132 ± 17	138 ± 18	0.009
Diastolic BP	83 ± 13	87 ± 15	0.077	82 ± 11	80 ± 12	0.271
Hypertension	67 (62%)	65 (74.7%)	0.084	55(55.6%)	65 (65.7%)	0.191
Dyslipidemia	55 (50.9%)	58 (66.7%)	0.039	50(50.5%)	69 (69.7%)	0.009
Neutrophil Count	3.8 ± 1.2	4 ± 1.3	0.392	3.9 ± 1.2	4.3 ± 1.5	0.037
Lymphocyte Count	1.8 ± 0.7	1.9 ± 0.7	0.87	2 ± 0.7	1.9 ± 0.6	0.239

^1 ^Microarray cohort analyses are restricted to those whose arrays passed QC analysis (195/198 for CATHGEN and 198/210 for the PREDICT samples)

**Figure 2:**
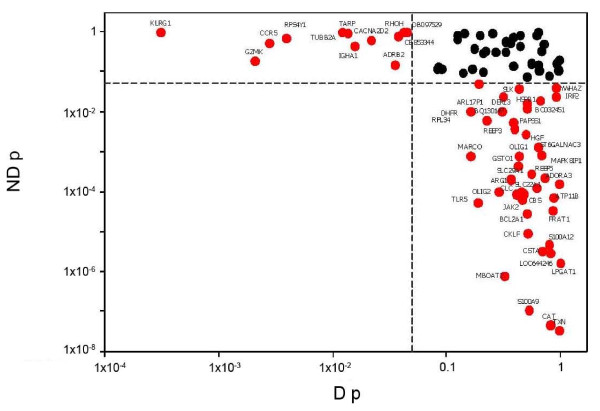
**RT-PCR analysis of diabetic status impact on significant genes from CATHGEN microarray analysis**. Significance of individual genes selected from the CATHGEN microarray cohort in non-diabetic (ND) and diabetic (D) patients is shown. The sex/age adjusted p values from a CAD logistic regression analysis in each subset are plotted (log scale). Significant p values (<0.05) are indicated in red with gene symbols, non-significant ones in black.

A total of 2,438 genes showed significant CAD association (p < 0.05) in the 195 subject case: control analysis from the CATHGEN cohort (Figure 1). Analysis of the effect of clinical factors on gene expression showed diabetes as the most significant (p = 0.0006, Additional file 2). Based on statistical significance and biological relevance, 88 genes (Additional file 2) were selected for RT-PCR analysis on these same samples. CAD-gene expression analysis in non-diabetic and diabetic subsets (N = 124 and 71, respectively), showed 42 and 12 significant genes, respectively (p < 0.05), with no intersection (Figure 2). Further work was thus limited to non-diabetics.

**Figure 3:**
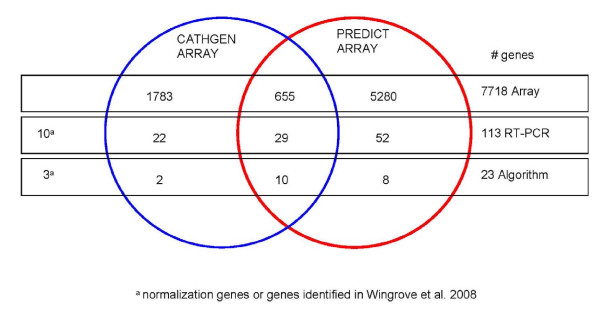
**Venn diagram of microarray, RT-PCR, and algorithm gene sources**. A total of 7718 genes were identified, 2438 and 5935, respectively, from the CATHGEN and PREDICT microarray analyses, with an intersection of 655 genes. For the 113 RT-PCR genes, 52 were from PREDICT, 22 from CATHGEN, and 29 from both; 10 were either normalization genes or from previous studies [13]. The final algorithm contained 20 informative genes: 10 from both microarray studies, 8 PREDICT alone, and 2 CATHGEN alone.

**Figure 4:**
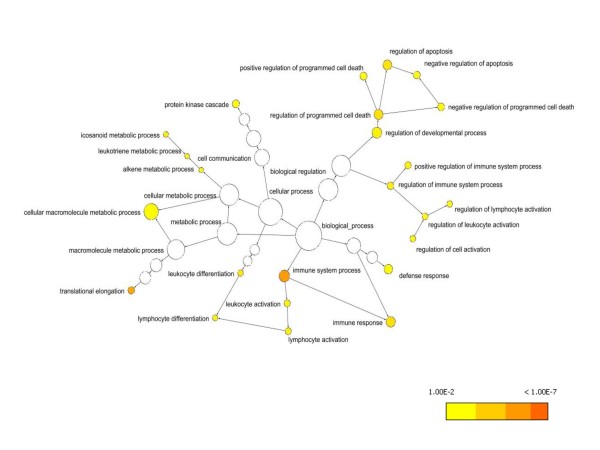
**Gene ontology analysis of 655 CAD genes identified from microarray studies**. The 655 CAD genes identified were analyzed using the BINGO algorithm to ascertain significant biological processes. Significant processes (p < 0.01 after FDR correction) are colored with the gradient of p values reflected in the colors as indicated, and the biological process annotated. A total of 55 processes were significant in this analysis at p < 0.05.

Microarray CAD gene discovery on 210 PREDICT non-diabetic patient samples used a paired case: control experimental design, to reduce confounding effects of age, sex and microarray batch processing. CAD analysis on the 99 case: control pairs which passed quality metrics yielded 5,935 significant genes (p < 0.05) with 655 genes in common with the CATHGEN results (Figure 3, Additional File 2). Gene Ontology (GO) analysis of these 655 genes identified 55 significant, overrepresented biological process terms (adjusted p < 0.05, Figure 4, Additional File 2), largely reflecting inflammation, immune cell differentiation, cell death and apoptosis. The molecular and cellular ontologies showed enrichment of 3 and 10 terms respectively, including caspase activity and ribosomal function.

### Gene selection

A total of 113 genes (Table 2) were selected by statistical significance, biological relevance, and prior association with CAD from RT-PCR gene expression measurements in the 640 patient PREDICT algorithm development cohort (Figure 1, Table 3). Known cell-type specific markers, those correlated with cell counts in PREDICT, and candidate normalization genes, were also represented.

**Table 2:** Genes evaluated by RT-PCR in the algorithm development cohort

Gene Symbol	MicroArray Evidence^1^	Cell-Type^2^	Cluster	Metagene Term	Algorithm Term^3^
DDX18	3		1.1		
SSRP1	3		1.2		
CCT2	3	2	1.3		
RPL28	N	2	1.4	Norm	2b
XIST	2	1,4,5	1.5		
RASSF7	3		1.6		
PKD1	3		1.7		
AGPAT5	3	2,7	1.8		
GLS	3		1.9		
TMC8	3		1.10	1	3b,4b
RPS4Y1	2	3	1.11		
KLF12	3	4	1.12		
LCK	2,3	3,4,8	1.13		
CD3D	2,3	3,4,8	1.14	1	3b,4b
AES	3		1.15		
ZAP70	3	3,4,8	1.16		
CD81	3	7,8	1.17		
QDPR	3	2,5	1.18		
FXN	2	2	1.19		
CORO2A	3		1.20		
TCEA1	3	7	1.21		
KMO	3	5,7	2.1		
TLR7	3	5	2.2		
RHOC	3		2.3		
CX3CR1	3	6,8	2.4		
IL11RA	1,2	3,4	3.1		
IL7R	1,2,3	3,4,8	3.2	3	
FAIM3	2,3	3,4,7	3.3		
TCF7	2,3	3,4,8	3.4	3	
CD79B	2,3	7	3.5	2	4a
SPIB	2,3	2,5,7	3.6	2	4a
CD19	3	5,7	3.7		
BLK	3	5,7	3.8		
PI16	2		3.9		
LRRN3	3	3,4	3.10	4	
HNRNPF	N		4.1	Norm	5b,6b
TFCP2	N		4.2	Norm	5b,6b
ACBD5	3		4.3		
DIAPH1	3		4.4		
CD37	3	7	4.5		
PLAGL2	3	1	4.6		
SRA1	3		5.1		
CD300A	2	8	5.2		
ELMO2	3	5,8	5.3		
CD33	2	1,6	6.1		
CSPG2	1,2		6.2		
CAT	2	2,5	6.3		
NOD2	1,3	1,6	6.4		
KCNMB1	2		6.5	5	
TCF7L2	3	1,6,8	6.6	5	
PDK4	3		6.7	5	
TBC1D8	3	1,5,6	6.8		
NR4A1	3	5	7.1		
CDKN1C	3	6,8	7.2		
C2	2		7.3		
CLC	2	1,2	8.1	6	
OLIG2	2		8.2		
ADORA3	2		8.3	6	
MMD	1,2,3	7	9.1		
HIST1H2AE	1,3	4,7	9.2	7	
AMFR	2		10.1		
CD34	N	2	10.2		
A_24_P128361 (AF289562)	3		11.1	8	5a
CD248	2,3	4	11.2		
KLRC4	2	4,8	12.1	9	3a
TARP	2,3	4,8	12.2		
CCR5	2	4,5	12.3		
CD8A	1	3,4,8	12.4		
SLAMF7	2	5,8	12.5	9	3a
KLRC2	2	3,4,8	12.6		
PRSS23	2	8	12.7		
NCAM1	N	8	12.8		
TNFRSF10C	3		13.1	11	1b
IL8RB	1,3	1,6,8	13.2	11	1b
TLR4	3	1,6	13.3	11	1b
NAMPT	3	1,5,6	13.4		
AQP9	3	1,6	13.5	10	2c
S100A8	1,2,3	1,5,6	13.6	12	2a
NCF4	2,3	1,6	13.7	10	2c
GLT1D1	1,2,3		13.8		
TXN	2,3	2,5	13.9		
GABARAPL1	3		13.10		
SIRPB2	1,3		13.11		
TRPM6	3		13.12		
CD93	1,2,3	1,5,6	13.13		
ASPRV1	3		13.14		
ALOX5AP	2,3	5	13.15		
BCL2A1	1,2,3	1,6,8	13.16		
F11R	3		14.1		
PTAFR	3	1,6	14.2		
H3F3B	3	7	14.3		
TYROBP	2,3	1,6,8	14.4		
NCF2	3	1,5,6	14.5		
KCNE3	2,3	1,6	14.6	11	1b
LAMP2	2,3	1	14.7		
PLAUR	3	1,6	14.8		
CD14	1	1,5,6	14.9		
HK3	1,2	1,6,8	14.10		
IL18	1		14.11		
RGS18	1,2	1,6	15.1		
BMX	2,3		16.1		
MMP9	2,3		16.2		
S100A12	1,2,3	1,5,6	16.3	12	2a
CLEC4E	2,3		16.4	12	2a
CLEC4D	2,3	1,6	16.5		
CASP5	2,3		16.6	13	1a
TNFAIP6	2,3	1	16.7	13	1a
IL18RAP	1,3	3,4,8	16.8	13	1a
ARG1	2,3		17.1	14	
HP	1	1,2	17.2		
CBS	2,3		17.3	14	
AF161365	3		17.4	15	6a
ALAS2	CN		18.1		

^1 ^Microarray Evidence: 1 = Wingrove et al, 2 = CATHGEN, 3 = PREDICT, CN = cell-type specific normalization gene, N = global normalization gene

^2 ^Cell Type: 1 = CD33+ (myeloid), 2 = CD34+(hematopoetic precursor), 3 = CD4+, (T cell) 4 = CD8+ (T cell), 5 = Dendritic, 6 = CD14+ (monocyte), 7 = CD19+ (B cell), 8 = CD56+ (natural killer cell).

^3 ^For algorithm term identification, genes in the numerators are listed as Na whereas those in the denominators are Nb.

**Table 3:** PREDICT algorithm development cohort clinical and demographic characteristics^1^

Variable	Controls (N = 410)	Cases (N = 230)	p.value
Sex (%Male)	193 (47.1%)	180 (78.3%)	<.001
Age (yrs)	57 ± 12	64 ± 11	<.001
Caucasian	347 (84.6%)	210 (91.3%)	0.022
BMI	31 ± 8	30 ± 6	0.348
Current Smoker	87 (21.2%)	45 (19.6%)	0.693
Systolic BP	133 ± 18	138 ± 18	<.001
Diastolic BP	80 ± 12	80 ± 11	0.944
Hypertension	248 (60.5%)	167 (72.6%)	0.003
Dyslipidemia	225 (54.9%)	170 (73.9%)	<.001
Neutrophil Count	4 ± 1.2	4.3 ± 1.4	0.054
Lymphocyte Count	2 ± 0.6	1.9 ± 0.6	0.007
Chest Pain Category			.0004
Asymptomatic	141 (35.4%)	90 (39.6%)	
Atypical	56 (14.0%)	29 (12.8%)	
Non-Anginal	137 (34.3%)	47 (20.7%)	
Typical	65 (16.3%)	61 (26.9%)	

^**1 **^Clinical and demographic characteristics for the 640 PREDICT algorithm development cohort are shown.

### Analysis of algorithm development cohort: clinical and gene expression factors

The most significant clinical/demographic factors for CAD association were age, sex, chest pain type and neutrophil count. Age and sex were independent risk factors for CAD (Table 3) and showed significant gene expression correlation. Chest pain type was also a significant independent risk factor (p = 0.0004), especially in men, but was gene expression independent. Neutrophil count was significantly correlated (positively or negatively) to expression of 93 of 113 RT-PCR genes, and was significantly associated with CAD in men (p = 0.049) but not women (p = 0.77). Neutrophil-associated genes showed both up and down regulation with CAD status, whereas lymphocyte-associated genes were generally down-regulated. There was significant gender-specific regulation of neutrophil correlated genes (men 40/42 genes up-regulated, women, 41/42 down-regulated) whereas lymphocyte gene down-regulation was gender independent.

**Figure 5:**
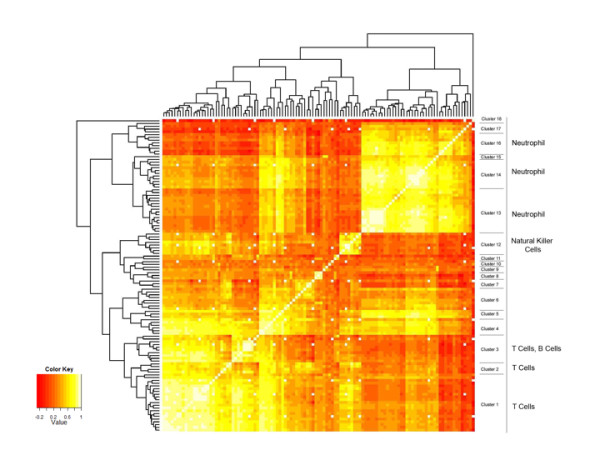
**Heat-Map representation of Hierarchical Clustering Results on 113 RT-PCR Genes**. Clusters were generated by hierarchical clustering yielding 20 groups of correlated. Clusters were annotated as to cell type expression using BioGPS (http://www.biogps.gnf.org). Extent of correlation is indicated by color as shown in the bar.

Hierarchical clustering of the 113 PCR genes resulted in 18 correlated clusters (Figure 5, Table 2), a significant fraction of which could be mapped to cell-type specific gene expression groups, with finer correlation substructure within the lymphocyte and neutrophil associated genes. There were 3 lymphocyte subgroups representing T-cells (clusters 1,2,3), B-cells (cluster 3) and NK cells (cluster 12). Three neutrophil subgroups were also identified: previously described neutrophil genes (IL8RB, S100A8, S100A12, TXN, BCL2A1; cluster 13, 16); newly identified up-regulated neutrophil genes (CLEC4E, CASP5, TNFAIP6; cluster 16) and down-regulated neutrophil genes (KCNE3, TLR4, TNFRSF10C; clusters 13, 14) [13]. Cluster 8 appears to be eosinophil specific. The 26 genes in clusters 4-7 and 9-11 did not have clear cell-type association.

### Algorithm derivation and performance

**Figure 6:**
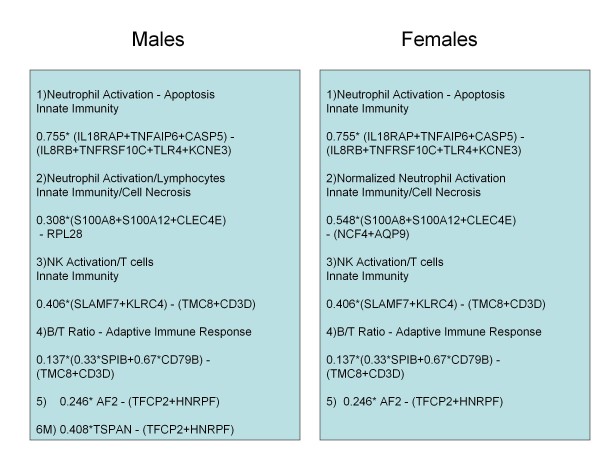
**Schematic of the final algorithm structure and genes**. The algorithm consists of overlapping gene expression functions for men and women with a sex-specific linear age function for the former and a non-linear age function for the latter. The genes in each term and their weights are shown. For the gene expression components, 16/23 genes in 4 terms are gender independent: Term 1 - neutrophil activation and apoptosis, Term 3 - NK cell activation to T cell ratio, Term 4, B to T cell ratio, and Term 5 -AF289562 expression normalized to TFCP2 and HNRPF. In addition, Term 2 consists of 3 sex-independent neutrophil/innate immunity genes (S100A8, S100A12, CLEC4E) normalized to overall neutrophil gene expression (AQP9, NCF4) for women and to RPL28 (lymphocytes) for men. The final male specific term is the normalized expression of TSPAN16. Algorithm score is calculated as described (Additional file 1).

Based on gene expression correlation clustering and cell-type analyses, 15 meta-genes and 3 normalization genes were defined as inputs for model variable selection (Table 2, Figure 6). Selection by the LASSO method and further weight penalization by Ridge regression resulted in the final, locked algorithm, comprising 20 CAD-associated genes and 3 normalization genes in 6 meta-gene terms (Figure 6), where each term represents a ratio of meta-genes or meta-gene to normalization genes. The algorithm score is calculated as described (Additional file 1) and was defined as the predicted regression model value.

**Figure 7:**
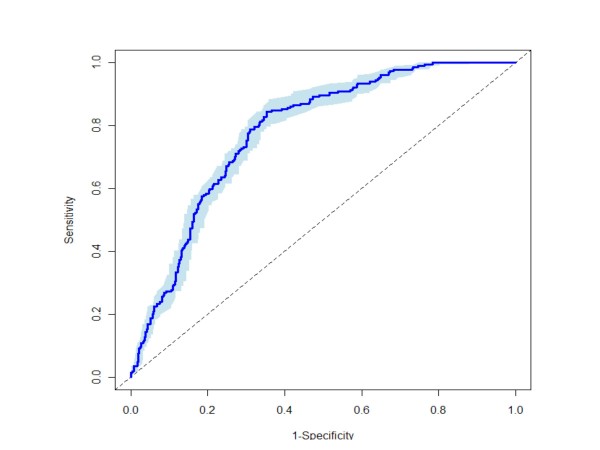
**ROC analysis of final algorithm**. The cross-validated ROC curve for the final algorithm in the algorithm development cohort is shown. The AUC is 0.77 ± 0.04.

The estimated cross-validated algorithm AUC in ROC analysis in the PREDICT development set was 0.77 (95% CI 0.73 to 0.81) (Figure 7); prospective validation in an independent PREDICT validation set of 526 patients (192 cases, 334 controls) yielded an AUC of 0.70 (95% CI = 0.65 to 0.75) [6].

## Discussion

This study presents gene discovery from microarrays and development from a large RT-PCR data set of a whole blood derived RT-PCR based gene-expression algorithm for assessment of obstructive CAD likelihood in non-diabetic patients, which was subsequently validated in an independent patient set [6].

The limitation to non-diabetic patients was due to the significant differences observed in PCR-based technical replication of the initial microarray experiment from the CATHGEN cohort, where both diabetic and non-diabetic patients were included (Figure 2). This effect could be due to differences in the pathophysiology of CAD in diabetics, as has been observed at the plaque composition level, [21] or due to diabetic medications, some of which modulate gene expression and affect cardiovascular disease [22].

A number of methodological steps deserve highlighting: first, we interrogated whole blood samples from more than 1,000 patients; second, we developed and used an automated and high reproducible RNA extraction process for the PREDICT samples; third, for the PREDICT work we also used core laboratory determined quantitative coronary angiography to define maximum percent stenosis and case: control status and fourth, we used ratios of correlated gene sets or meta-genes as building blocks for algorithm development. These methodological approaches enhanced the power of the PCR algorithm development set to investigate the relationship between CAD, clinical factors, and gene expression.

The relationships between age, sex, CAD, and gene expression are complex. Increasing age and male sex are well-known risk factors for CAD, which affect gene expression in circulating cells [23,24]. The majority of genes measured by RT-PCR in this study correlated with lymphocyte or neutrophil fraction. Lymphocyte-associated gene expression decreases with CAD in a sex-independent fashion, consistent with decreased lymphocyte counts being correlated with increased cardiovascular risk [14]. In contrast, neutrophil-associated genes display significant sex-specific expression differences with CAD: in men 95% of the neutrophil genes were up-regulated whereas 98% were down-regulated in women, consistent with increased granulocyte counts in men being associated with higher CAD risk, with lesser effects in women [25,26].

### Biological significance of algorithm terms

The use of correlated meta-genes as building blocks for the algorithm is significantly reflective of gene expression cell-type specificity. The algorithm genes are expressed selectively in multiple types of circulating cells including neutrophils, NK cells, B and T-lymphocytes, [27], supporting roles for both adaptive and innate immune responses in atherosclerosis [10].

A role for neutrophils in both the early and later stages of atherogenesis has recently been suggested, especially in connection with hyperlipidemia [28,29]. Algorithm term 1 is a ratio of neutrophil expressed meta-genes that are up and down regulated with CAD (Figure 6). This term may particularly reflect neutrophil apoptosis, as Caspase-5 is increased with CAD, whereas TNFRSF10C, an anti-apoptotic decoy receptor of TRAIL, is decreased [30]. Term 2 genes up-regulated with CAD are also expressed largely by neutrophils and likely reflect both innate immune activation, (S100A8 and S100A12), [31] and a cellular necrosis response (CLEC4E) [32]. S100A8 and S100A12 are up-regulated in chronic inflammatory conditions, including asthma, rheumatoid, and inflammatory arthritis, perhaps reflecting a more general pathophysiological signal, consistent with increased CAD in disorders such as rheumatoid arthritis [33,34].

Term 2 is highly correlated with the signature previously identified by us [13] and includes the most significant gene from that work, S100A12. This term is normalized in a sex-specific manner, perhaps reflecting sex-specific differences in the significance of neutrophil counts in CAD and MI [26]. In men normalization to RPL28 which is strongly expressed in lymphocytes, reflects the neutrophil to lymphocyte ratio, which is prognostic for death or MI in a CAD population [14]. In women normalization to AQP9 and NCF4, two CAD insensitive neutrophil genes, permits assessment of neutrophil up-regulation of the S100s and CLEC4E, independent of neutrophil count.

Term 3 consists of 2 NK cell receptors, SLAMF7 and KLRC4, normalized to T-cell specific genes (TMC8 and CD3D). SLAMF7 may specifically activate NK cell function, while inhibiting B and T cells [35]. KLRC4 is also likely involved in NK cell activation [36]. NK cells have been associated with atherosclerosis in both mouse models and humans, and reduced lymphocyte counts associated with cardiac events [14,37].

Term 4 is a gene expression based measure of the B/T-cell ratio. The roles of both T and B cells in atherosclerosis development are complex; mouse models have shown B cells to be both athero-protective and more recently, atherogenic [38-40]. In this study apparent upregulation of B-cell specific genes is correlated with CAD, perhaps supporting the latter. The last two terms, based on AF289562 (AF2) and TSPAN16 are genes of unknown function that will require further work to clarify their role in atherosclerosis.

Previous work by Sinnaeve and coworkers also examined peripheral blood gene expression in a coronary disease population [12]. As noted by these authors, there is little overlap between their genes and the signatures identified in our previous study [13] or this one. A number of significant differences in the study populations (restricted age range, combining two sex specific cohorts) in their study may have contributed to this. In addition, there are differences in both RNA isolation methodology and microarray platforms. Further work is needed to resolve these issues.

### Algorithm development

For algorithm development, as described above, we used an approach that minimized the effect of any single gene by using meta-genes as building blocks [18,41] Penalized stepwise logistic regression (LASSO) selected significant meta-genes from a 640 patient data set which greatly exceeded the number of candidate variables (15 meta-genes), reducing the likelihood of over-fitting. Further, in order to minimize over-weighting of individual terms, meta-gene coefficients were penalized using Ridge regression. An alternative approach would have been to use a combined two-step shrinkage method such as the elastic net [42]. Although correlations with specific cell types was a key observation, recent methodologies described for deconvoluting gene expression data sets from complex cell mixtures might have led to improved results [43].

The cross-validated model AUC was 0.77 (95% CI 0.73 to 0.81), suggesting that the algorithm score was a significant CAD predictor. A decrease to an AUC of 0.70, with overlapping confidence intervals (95% CI = 0.65 to 0.75), was observed in the independent validation set [6]. This decrease may reflect an over-optimistic cross-validation estimate, as we did not re-select terms during each iteration. Ultimately, the validation results provide the most informative measure of a model's prediction accuracy.

### Limitations

Although this is one of the largest studies examining gene expression in peripheral blood in CAD patients and has yielded a specific algorithm for the assessment of CAD status, it has several limitations.

From a clinical perspective, diabetics and patients with known chronic inflammatory disorders were excluded. The differences observed between diabetics and non-diabetics with CAD could be due to differences in the molecular pathophysiology of the disease, medications, or some combination of the two. In addition, although race was not an independent risk factor after adjustment for age and sex, the number of minority patients was low, so conclusions with respect to them are significantly underpowered. The use of a dichotomous angiographic endpoint does not account for variations in disease burden or external remodeling, and is not a measure of ischemia. Finally, the contribution of atherosclerosis in other vascular beds is outside the scope of this study, but may be important in asymptomatic high-risk individuals.

From a cellular and gene expression perspective, the relative ease of obtaining peripheral blood cell RNA is counter-balanced by not directly interrogating changes in the diseased vascular wall. Another complementary approach could be to examine secreted proteins in the blood that might reflect endothelial or vascular dysfunction. Finally, given the chronic nature of atherosclerotic disease, it is likely the gene expression signature observed reflects a response to disease rather than the underlying cause.

## Conclusions

Using a series of microarray and RT-PCR data sets, comprising more than 1,000 patients, we have derived an algorithm, consisting of the expression levels of 23 genes, sex, and age, which can assess the likelihood of obstructive CAD in non-diabetic patients.

## Abbreviations

CAD: coronary artery disease; MI: myocardial infarction; MPI: myocardial perfusion imaging; RT-PCR: real-time polymerase chain reaction; QCA: quantitative coronary angiography; ROC: receiver-operator characteristics; AUC: area under the curve.

## Competing interests

This work was funded by CardioDx, Inc. MRE, JAW, PB, SED, and SR are employees of CardioDx, Inc and have equity interests and/or stock options in CardioDx. WGT is a former employee and has equity or stock options in CardioDx. SR, MRE, JAW, PB and WGT have filed patent applications on behalf of CardioDx, Inc. WEK reports research support from CardioDx. EJT is supported in part by the Scripps Translational Science Institute Clinical Translational Science Award (NIHU54RR02504-01). AJL reports funding from CardioDx to complete the QCA studies reported herein. RSS, SZ, RW, JM, and NT report no conflicts of interest with respect to the contents of this manuscript.

## Authors' contributions and information

MRE, PB, JAW, SED, WGT, SR, SZ, GG, AL, WEK, RSS, and EJT contributed to the Conception, Design, and Data Analysis for this work as well as drafting and approving the final manuscript. SE, NT, RT, and JM helped critically revise the manuscript and all authors approved the final version. The remaining PREDICT investigators are listed in the Acknowledgements.

## Pre-publication history

The pre-publication history for this paper can be accessed here:

http://www.biomedcentral.com/1755-8794/4/26/prepub

## Supplementary Material

Additional file 1 **Algorithm Score Calculation and Transformation**. Cell fractionation and cell specific gene expression analysis. Click here for file

Additional file 2 **Data Tables**. Table S1 - Significance of Clinical Variables in CATHGEN gene discovery cohort. Table S2 - Significance of RT-PCR results for the 88 genes tested in the CATHGEN discovery cohort, in the non-diabetic and diabetic subsets. Table S3 - The 655 genes identified in both the CATHGEN and PREDICT discovery microarray experiments. Table S4 - The significant biological process, cellular compartment and molecular function ontologies from GO analysis of the 655 genes. Click here for file
