# Supplementary material for: Development of a blood-based gene expression algorithm for assessment of obstructive coronary artery disease in non-diabetic patients
Source: BMC Med Genomics. 2011 Mar 28;4:26. doi: 10.1186/1755-8794-4-26 (PMC3072303; doi:10.1186/1755-8794-4-26)
Supplement: Additional file 1 — Algorithm Score Calculation and Transformation. Cell fractionation and cell specific gene expression analysis. [file 1755-8794-4-26-S1.DOC]

**Supplementary Methods**

**Algorithm Calculation and Transformation**

**Data Preprocessing and QC Steps**

1. Compute median of triplicate wells for each algorithm gene/sample
   1. If one well has a no call, take the median of the two remaining wells
   2. If two or three wells have a no call, the algorithm gene receives a no call for that sample
2. If AF161365 (TSPAN16) receives a no call, impute the value of 38 as the median value for that gene.
3. If any algorithm gene other than AF161365 receives a no call, the sample fails for *Missing Gene Cp*. None of the 640 samples in Algorithm Development would fail this metric.
4. Compute the median of the algorithm gene SD’s, excluding AF161365. If this value is greater than .15, the sample fails for *High Replicate SD*.
5. For each algorithm gene i, floor the Cp value by replacing values less than GL_i_ with GL_i_ This value represents the 1^st^ percentile of Cp for that gene in the Algorithm Development set.
6. For each algorithm gene i, ceiling the Cp value by replacing values greater than GU_i_ with GU_i_. This value represents the 99^th^ percentile of Cp for that gene in the Algorithm Development set.
7. For each algorithm gene i, compute the absolute value of the difference between its Cp value and GM_i_, where GM_i_ represents the median Cp for that gene in the Algorithm Development set. Sum this value across the algorithm genes (excluding AF161365). If the sum is greater than 27.17, the sample fails for *Expression Profile Out of Range*. 27.17 represents the largest value of this metric within the Algorithm Development set.

In certain cases, an algorithm score will not be calculated for a subject. Reasons for this include low PAX tube blood volume, lab QC failure, etc. The frequency of occurrence of these failures will be tabulated, though these subjects will not be included in the analysis set. Subjects with missing Diamond Forrester scores will not be included in the analysis set.

**Algorithm Calculation**

1. Define Norm_1_ = RPL28
2. Define Norm_2_ = (.5*HNRPF + .5*TFCP2)
3. Define NK_up_ = (.5*SLAMF7 + .5*KLRC4)
4. Define T_cell_ = (.5*CD3D + .5*TMC8)
5. Define B_cell_ = (2/3 *CD79B + 1/3 * SPIB)
6. Define Neut = (.5*AQP9 + .5*NCF4)
7. Define N_up_ = (1/3 * CASP5 + 1/3*IL18RAP + 1/3*TNFAIP6)
8. Define N_down_ = (.25*IL8RB + .25*TNFRSF10C + .25*TLR4 + .25*KCNE3)
9. Define SCA_1_ = (1/3*S100A12 + 1/3*CLEC4E + 1/3*S100A8)
10. Define AF_2_ = AF289562
11. Define TSPAN = 1 if (AF161365-Norm2 > 6.27 or AF161365=NoCall), 0 otherwise
12. Define SEX= 1 for Males, 0 for Females
13. Define Intercept
    1. For Males, INTERCEPT = 2.672 + 0.0449*Age
    2. For Females, INTERCEPT = 1.821 + 0.123*(Age-60), if negative set to 0
14. Define Score = INTERCEPT – 0.755 *( N_up_ - N_down_) – 0.406*( NK_up_ - T_cell_) – 0.308 *SEX*( SCA_1_- Norm_1_)- 0.137* ( B_cell_- T_cell_)- 0.548 *(1-SEX)*( SCA_1_- Neut)- 0.482 *SEX*(TSPAN)- 0.246 *( AF_2_- Norm_2_)

**Score Transformation**

The endpoint analyses defined were performed using raw algorithm scores. For clinical reporting purposes, as well as ease of presentation, raw scores may be transformed into a transformed score with a scale designed for ease of clinical use as follows:

Input is Raw Score

If Raw Score< -2.95, set RawScore = -2.95

If Raw Score> 1.57, set RawScore = 1.57

Raw Score = 2.95 + RawScore

Final Score = RawScore*40/4.52

Round Final Score up to nearest integer

If Final Score is greater than 40, set to 40

If Final Score is less than 1, set to 1

Value obtained is the Final Transformed Score

**Estimation of Score Variability**

A total of 41 replicate samples were tested from a large PAX blood pool.  The standard deviation of the raw score for these replicates was .13.  The confidence interval around a given raw score was then the raw score plus or minus 1.96*.13.  The upper and lower bounds of this confidence interval were linearly transformed to the 0 to 40 scale, and then transformed to a confidence interval around the likelihood using the score to likelihood function described above.
