# Supplementary material for: Development of a blood-based gene expression algorithm for assessment of obstructive coronary artery disease in non-diabetic patients
Source: BMC Med Genomics. 2011 Mar 28;4:26. doi: 10.1186/1755-8794-4-26 (PMC3072303; doi:10.1186/1755-8794-4-26)
Supplement: Additional file 2 — Data Tables. Table S1 - Significance of Clinical Variables in CATHGEN gene discovery cohort. Table S2 - Significance of RT-PCR results for the 88 genes tested in the CATHGEN discovery cohort, in the non-diabetic and diabetic subsets. Table S3 - The 655 genes identified in both the CATHGEN and PREDICT discovery microarray experiments. Table S4 - The significant biological process, cellular compartment and molecular function ontologies from GO analysis of the 655 genes. [file 1755-8794-4-26-S2.DOC]

**Methods**

**Algorithm Calculation and Transformation**

**Data Preprocessing and QC Steps**

1. Compute median of triplicate wells for each algorithm gene/sample
   1. If one well has a no call, take the median of the two remaining wells
   2. If two or three wells have a no call, the algorithm gene receives a no call for that sample
2. If AF161365 (TSPAN16) receives a no call, impute the value of 38 as the median value for that gene.
3. If any algorithm gene other than AF161365 receives a no call, the sample fails for *Missing Gene Cp*. None of the 640 samples in Algorithm Development would fail this metric.
4. Compute the median of the algorithm gene SD’s, excluding AF161365. If this value is greater than .15, the sample fails for *High Replicate SD*.
5. For each algorithm gene i, floor the Cp value by replacing values less than GL_i_ with GL_i_ This value represents the 1^st^ percentile of Cp for that gene in the Algorithm Development set.
6. For each algorithm gene i, ceiling the Cp value by replacing values greater than GU_i_ with GU_i_. This value represents the 99^th^ percentile of Cp for that gene in the Algorithm Development set.
7. For each algorithm gene i, compute the absolute value of the difference between its Cp value and GM_i_, where GM_i_ represents the median Cp for that gene in the Algorithm Development set. Sum this value across the algorithm genes (excluding AF161365). If the sum is greater than 27.17, the sample fails for *Expression Profile Out of Range*. 27.17 represents the largest value of this metric within the Algorithm Development set.

In certain cases, an algorithm score will not be calculated for a subject. Reasons for this include low PAX tube blood volume, lab QC failure, etc. The frequency of occurrence of these failures will be tabulated, though these subjects will not be included in the analysis set. Subjects with missing Diamond Forrester scores will not be included in the analysis set.

**Algorithm Calculation**

1. Define Norm_1_ = RPL28
2. Define Norm_2_ = (.5*HNRPF + .5*TFCP2)
3. Define NK_up_ = (.5*SLAMF7 + .5*KLRC4)
4. Define T_cell_ = (.5*CD3D + .5*TMC8)
5. Define B_cell_ = (2/3 *CD79B + 1/3 * SPIB)
6. Define Neut = (.5*AQP9 + .5*NCF4)
7. Define N_up_ = (1/3 * CASP5 + 1/3*IL18RAP + 1/3*TNFAIP6)
8. Define N_down_ = (.25*IL8RB + .25*TNFRSF10C + .25*TLR4 + .25*KCNE3)
9. Define SCA_1_ = (1/3*S100A12 + 1/3*CLEC4E + 1/3*S100A8)
10. Define AF_2_ = AF289562
11. Define TSPAN = 1 if (AF161365-Norm2 > 6.27 or AF161365=NoCall), 0 otherwise
12. Define SEX= 1 for Males, 0 for Females
13. Define Intercept
    1. For Males, INTERCEPT = 2.672 + 0.0449*Age
    2. For Females, INTERCEPT = 1.821 + 0.123*(Age-60), if negative set to 0
14. Define Score = INTERCEPT – 0.755 *( N_up_ - N_down_) – 0.406*( NK_up_ - T_cell_) – 0.308 *SEX*( SCA_1_- Norm_1_)- 0.137* ( B_cell_- T_cell_)- 0.548 *(1-SEX)*( SCA_1_- Neut)- 0.482 *SEX*(TSPAN)- 0.246 *( AF_2_- Norm_2_)

**Score Transformation**

The endpoint analyses defined were performed using raw algorithm scores. For clinical reporting purposes, as well as ease of presentation, raw scores may be transformed into a transformed score with a scale designed for ease of clinical use as follows:

Input is Raw Score

If Raw Score< -2.95, set RawScore = -2.95

If Raw Score> 1.57, set RawScore = 1.57

Raw Score = 2.95 + RawScore

Final Score = RawScore*40/4.52

Round Final Score up to nearest integer

If Final Score is greater than 40, set to 40

If Final Score is less than 1, set to 1

Value obtained is the Final Transformed Score

**Estimation of Score Variability**

A total of 41 replicate samples were tested from a large PAX blood pool.  The standard deviation of the raw score for these replicates was .13.  The confidence interval around a given raw score was then the raw score plus or minus 1.96*.13.  The upper and lower bounds of this confidence interval were linearly transformed to the 0 to 40 scale, and then transformed to a confidence interval around the likelihood using the score to likelihood function described above.

**Fractionation of Whole Blood Cells for Cell-type Specific Gene Expression Measurements**

Cell fractionation was performed on fresh blood collected in EDTA tubes. 120 ml blood pooled from 4 different donors was 1:1 diluted with 1X PBS. 15% of the blood was used for granulocyte isolation by density centrifugation and 85% of the blood was used for PBMC isolation prior to T cells, B cells, NK cells and monocytes fractionation.

**PBMC isolation**

PBMC was isolated by density centrifugation. 20 ml diluted blood was layered on 20ml Histopaque 1077 (Sigma Cat No.10771) in 50ml conical tubes and was centrifuged at room temperature for 30 min at 400Xg. The PBMC layer was carefully aspirated into new tubes and washed with 1X PBS twice and centrifuged at 200Xg for 10 min. The washed PBMC was re-suspended in cold buffer1 (1XPBS, 0.1%BSA and 2mMEDTA) and stored on ice. 5% of the cells were lysed in RLT buffer (Qiagen RNeasy Mini kit, Cat No. 74104) for pre-selection RNA isolation.

**Granulocyte isolation**

Granulocytes (neutrophils, eosinophils, basophils) were purified by density centrifugation using two different density mediums. In 15ml conical tube, 3ml Hisopaque 1077 was layered on 3ml Histopaque 1119 (Sigma Cat No.11191) and 6ml of the diluted blood was then layered on Histopaque 1077. The tube was centrifuged at RT for 30min at 700Xg. The granulocyte layer was then aspirated into a new tube and washed twice. The pellet was re-suspended in RLT buffer for granulocyte RNA isolation.

**Positive cell isolation with magnetic beads**

The subsequent cell types (T cells, B cells, NK cells, monocytes) were positively selected from PBMC used the following reagents and the recommended procedures.

CD8+ T cells –­­ Dynal® CD8 positive isolation kit (Invitrogen Cat. No.113.33D)

CD3+ T cells – Dynabeads® CD3 (Invitrogen Cat. No.111.51D)

CD19+ B cells – Dynabeads® CD19 pan B (Invitrogen Cat. No.111.43D)

CD14+ Monocytes – Dynabeads® CD14 (monocytes/macrophages) (Invitrogen Cat. No.111.49D)

CD56+ NK cells– Dynabeads® Pan Mouse IgG (Invitrogen Cat. No.110.41) cross-linked with mouse anti-human CD56 antibodies (BD bioscience Cat No.556325)

Briefly, PBMC were incubated with antibody-coupled magnetic beads at 4^0^C for 20 min and washed 3 times with buffer 1 on the magnet. The selected cells were then re-suspended in RLT buffer for RNA isolation.

**RNA isolation**

The RNA samples in RLT buffer were purified using the Qiagen RNeasy Mini kit following manufacture’s instruction.

**Supplementary Tables**

Table S1. Significance of Clinical Variables in CATHGEN gene discovery set.

| Clinical Variable | p-value |
| --- | --- |
| Diabetes | 0.000560741 |
| Anti Hypertensive Use | 0.012462227 |
| HDL | 0.088459908 |
| Neutrophil Count | 0.129686671 |
| Antidiabetic Use | 0.140870844 |
| LDL | 0.146873756 |
| Total Cholesterol | 0.172382024 |
| WBC Count | 0.189994635 |
| Lipid Lowering Agent Use | 0.200078333 |
| Triglycerides | 0.207728761 |
| Diastolic BP | 0.21703689 |
| Chest Pain | 0.219704278 |
| Monocyte Count | 0.23769698 |
| Platelet Count | 0.238534146 |
| Smoker | 0.257352165 |
| Lymphocyte Count | 0.261169567 |
| Anticoagulant Use | 0.321044006 |
| Anti Inflammatory Use | 0.332101624 |
| Antiplatelet Use | 0.336359859 |
| Statin Use | 0.390097042 |
| Calcium Channel Blocker Use | 0.401676568 |
| Sex | 0.409669446 |
| Postmenopausal | 0.418849343 |
| Alcohol Use | 0.495208348 |
| NSAID Use | 0.536650232 |
| ACE Inhibitor Use | 0.687539195 |
| Vasodilator Use | 0.715979777 |
| Systolic BP | 0.716766737 |
| Antiarrhythmic Use | 0.763504492 |
| Salicylates | 0.805576705 |
| Beta Blocker Use | 0.819779733 |
| Hypertension | 0.834786056 |
| Black | 0.847458733 |
| Age | 0.984504316 |

Table S2. RT-PCR Results on CATHGEN cohort genes.

| Gene | Non-Diabetic p | Diabetic p |
| --- | --- | --- |
| KLRG1 | 0.933635139 | 0.000313584 |
| GZMK | 0.176629393 | 0.002075813 |
| CCR5 | 0.524551866 | 0.002796076 |
| RPS4Y1 | 0.641924002 | 0.003924492 |
| TUBB2A | 0.905726045 | 0.012164059 |
| TARP | 0.855579011 | 0.013579949 |
| IGHA1 | 0.427023322 | 0.015653596 |
| CACNA2D2 | 0.579670417 | 0.021884775 |
| ADRB2 | 0.14583996 | 0.035331896 |
| DB097529 | 0.739638806 | 0.037474362 |
| CB853344 | 0.924313185 | 0.042530621 |
| RHOH | 0.914493918 | 0.045421079 |
| GPR114 | 0.113792718 | 0.082926442 |
| RPS27A | 0.127518837 | 0.085484803 |
| CD3E | 0.114159341 | 0.090230797 |
| RELA | 0.800147639 | 0.124184492 |
| HDC | 0.611947115 | 0.124749411 |
| NR1D1 | 0.08855384 | 0.140309177 |
| RRN3 | 0.883475152 | 0.14306721 |
| MARCO | 0.000742446 | 0.162858627 |
| ARL17P1 | 0.009929764 | 0.163503477 |
| POLR2L | 0.110001621 | 0.169570816 |
| RPL10A | 0.372025559 | 0.176554229 |
| TLR5 | 5.31034E-05 | 0.187801635 |
| RPL34 | 0.047258313 | 0.194514225 |
| CARKL | 0.796426726 | 0.197876342 |
| DPM3 | 0.100527185 | 0.210155758 |
| C11orf2 | 0.279960963 | 0.21235462 |
| LIF | 0.319291 | 0.220377076 |
| DHFR | 0.005845519 | 0.227352382 |
| BU540282 | 0.855833364 | 0.253041264 |
| CDC42SE2 | 0.303933209 | 0.27279888 |
| OLIG2 | 9.8531E-05 | 0.291441723 |
| DERL3 | 0.009989003 | 0.311630921 |
| SLK | 0.022499454 | 0.315243668 |
| MBOAT2 | 7.53321E-07 | 0.32533079 |
| ST3GAL1 | 0.555439718 | 0.329090787 |
| FOLR3 | 0.293485861 | 0.330960224 |
| NDUFS7 | 0.510992855 | 0.362739986 |
| SLC29A1 | 0.000196258 | 0.370006714 |
| TCF7 | 0.139201093 | 0.384656786 |
| BQ130147 | 0.005433882 | 0.39124831 |
| SPSB2 | 0.710554126 | 0.392430072 |
| REEP3 | 0.003636115 | 0.39572088 |
| CBS | 8.54923E-05 | 0.414841711 |
| GSTO1 | 0.000439166 | 0.421164955 |
| VSIG4 | 0.03654483 | 0.436274059 |
| OLIG1 | 0.000739337 | 0.438928192 |
| RPL8 | 0.420798397 | 0.441110854 |
| CR609588 | 0.829179104 | 0.44827808 |
| ARG1 | 9.77852E-05 | 0.454989416 |
| JAK2 | 6.14999E-05 | 0.462535965 |
| CLC | 8.43913E-05 | 0.478209075 |
| PAPSS1 | 0.002660178 | 0.497255641 |
| HSPB1 | 0.011649931 | 0.503891496 |
| MPZL1 | 0.069994815 | 0.504344915 |
| BC032451 | 0.015738039 | 0.505628786 |
| BCL2A1 | 2.81815E-05 | 0.50979301 |
| CKLF | 8.76337E-06 | 0.515802792 |
| S100A9 | 1.04727E-07 | 0.5350388 |
| MAPK8IP1 | 0.000267919 | 0.558711324 |
| LOXL2 | 0.153997075 | 0.559866641 |
| GSTP1 | 0.802223179 | 0.622441442 |
| SLC22A1 | 0.000127897 | 0.626928629 |
| HGF | 0.001272015 | 0.63284641 |
| EPOR | 0.918974368 | 0.633466985 |
| ETFB | 0.143878666 | 0.645850919 |
| SSNA1 | 0.103788889 | 0.6470392 |
| IRF2 | 0.018278933 | 0.665824694 |
| ASMTL | 0.311592758 | 0.681691103 |
| ST6GALNAC3 | 0.000812432 | 0.686396961 |
| CSTA | 3.1114E-06 | 0.707081235 |
| SMN1 | 0.473451351 | 0.714837746 |
| REEP5 | 0.000215833 | 0.733733395 |
| FCGBP | 0.074075812 | 0.796385743 |
| S100A12 | 4.72256E-06 | 0.804439181 |
| CAT | 4.59232E-08 | 0.81384176 |
| LOC644246 | 2.85943E-06 | 0.820487985 |
| FRAT1 | 3.39803E-05 | 0.859050707 |
| ATP11B | 6.96563E-05 | 0.882770629 |
| LGALS1 | 0.039299421 | 0.918250705 |
| YWHAZ | 0.023358903 | 0.927846666 |
| MMD | 0.153204886 | 0.941639541 |
| CD33 | 0.101691174 | 0.950753885 |
| CD248 | 0.186672242 | 0.973814259 |
| ADORA3 | 0.000150846 | 0.975200559 |
| TXN | 3.22949E-08 | 0.99228328 |
| LPGAT1 | 1.58563E-06 | 0.995574922 |

Table S3. 655 Genes found in both CATHGEN and PREDICT Microarray Analyses

| Gene Symbol |
| --- |
| AA303143 |
| AA601031 |
| ABCC2 |
| ABHD2 |
| ABHD5 |
| ABLIM1 |
| ACO2 |
| ACOX1 |
| ACSL1 |
| ACTB |
| ACVR2B |
| ADA |
| ADNP |
| AF034187 |
| AF085968 |
| AF161353 |
| AF471454 |
| AI276257 |
| AIM1L |
| AK021463 |
| AK022268 |
| AK023663 |
| AK024956 |
| AK056689 |
| AK092942 |
| AK098835 |
| AK124192 |
| ALOX12 |
| ALOX5 |
| ALOX5AP |
| ALS2CR13 |
| AMBN |
| AMFR |
| AMICA1 |
| ANXA2 |
| ANXA3 |
| AOAH |
| AP1S2 |
| APBA2 |
| APBB1 |
| APEH |
| APH1A |
| APOBEC3G |
| APRT |
| AQP2 |
| AQP8 |
| ARG1 |
| ARHGAP24 |
| ARHGAP9 |
| ARHGDIA |
| ARID5B |
| ARPC1B |
| ASCL2 |
| ATG3 |
| ATP1B2 |
| ATP5D |
| ATP6V0B |
| ATP7B |
| AW076051 |
| AW579245 |
| AX721252 |
| AY003763 |
| AY062331 |
| A_23_P158868 |
| A_23_P335398 |
| A_23_P348587 |
| A_23_P44053 |
| A_24_P101960 |
| A_24_P144383 |
| A_24_P221375 |
| A_24_P238427 |
| A_24_P384604 |
| A_24_P417996 |
| A_24_P418712 |
| A_24_P745883 |
| A_24_P84408 |
| A_24_P916228 |
| A_24_P929533 |
| A_32_P28158 |
| A_32_P62137 |
| B2M |
| B4GALT5 |
| BACH2 |
| BAGE |
| BAZ1A |
| BBS2 |
| BC024289 |
| BC031973 |
| BC038432 |
| BC043173 |
| BC062739 |
| BC073935 |
| BCL2A1 |
| BCL3 |
| BCL6 |
| BCL7A |
| BG777521 |
| BI024548 |
| BI026064 |
| BM703463 |
| BMX |
| BOP1 |
| BQ365891 |
| BRF1 |
| BRI3 |
| BST1 |
| BTBD14A |
| BTNL8 |
| BU633383 |
| BX110908 |
| BYSL |
| C10orf54 |
| C11orf2 |
| C12orf35 |
| C14orf156 |
| C15orf38 |
| C16orf24 |
| C16orf57 |
| C1orf96 |
| C20orf24 |
| C20orf3 |
| C20orf77 |
| C2orf39 |
| C6orf129 |
| C6orf32 |
| C7orf34 |
| C8orf31 |
| C9orf19 |
| CALM3 |
| CAMKK2 |
| CAPNS1 |
| CASP4 |
| CASP5 |
| CBS |
| CCDC108 |
| CCDC92 |
| CCL3L3 |
| CCPG1 |
| CD200 |
| CD248 |
| CD302 |
| CD3D |
| CD3E |
| CD5 |
| CD58 |
| CD6 |
| CD7 |
| CD79B |
| CD93 |
| CD96 |
| CDKL5 |
| CDKN1A |
| CEACAM4 |
| CEBPB |
| CEBPD |
| CFLAR |
| CFP |
| CHI3L2 |
| CIB3 |
| CKLF |
| CLEC12A |
| CLEC2D |
| CLEC4D |
| CLEC4E |
| CLIC1 |
| CMTM2 |
| CNTNAP2 |
| COL14A1 |
| COMMD6 |
| COP1 |
| COX6B2 |
| COX6C |
| CPD |
| CR2 |
| CR593845 |
| CR610181 |
| CR613361 |
| CR613944 |
| CREB5 |
| CRIP1 |
| CRISPLD2 |
| CSF2RA |
| CSF2RB |
| CSTA |
| CTBP2 |
| CYB5D2 |
| CYP1A2 |
| CYP4F2 |
| CYP4F3 |
| CYP4F8 |
| DCXR |
| DDX11 |
| DDX3Y |
| DEDD2 |
| DEFA4 |
| DEK |
| DENND3 |
| DHRS3 |
| DHRS7B |
| DHRSX |
| DKFZP434B0335 |
| DKFZp434F142 |
| DKFZp547E087 |
| DOCK10 |
| DOCK8 |
| DOK3 |
| DPF3 |
| DPPA5 |
| DRAP1 |
| DUOX2 |
| DUSP13 |
| DUSP3 |
| DYNLT1 |
| ECH1 |
| ECHDC3 |
| EEF2 |
| EIF1AX |
| EIF2AK2 |
| EIF2C4 |
| EIF4B |
| EIF5A |
| EMP3 |
| EMR3 |
| ENST00000337102 |
| ENST00000360102 |
| ENTPD1 |
| ETS1 |
| EXOC6 |
| EXOSC6 |
| F5 |
| FAIM3 |
| FAM108A1 |
| FAM113B |
| FAM26B |
| FAM44A |
| FAU |
| FBXL5 |
| FCAR |
| FCER1A |
| FGD4 |
| FIBP |
| FKBP5 |
| FKBP9 |
| FLJ22662 |
| FLJ40092 |
| FNDC3B |
| FOS |
| FOXJ1 |
| FOXP1 |
| FPR1 |
| FRAT1 |
| FRAT2 |
| FRS2 |
| FRS3 |
| FTH1 |
| FXYD5 |
| FYB |
| GADD45GIP1 |
| GAMT |
| GBP2 |
| GCA |
| GLRX |
| GLT1D1 |
| GLUL |
| GMFG |
| GNB1 |
| GPA33 |
| GPBAR1 |
| GPC1 |
| GPD1 |
| GPR160 |
| GPR172A |
| GPR37L1 |
| GRB10 |
| GSTT1 |
| GTF2I |
| GYG1 |
| H2AFZ |
| H3F3A |
| HAL |
| HAP1 |
| HDAC4 |
| HDDC2 |
| HDGFL1 |
| HEBP2 |
| HIST1H2AC |
| HIST1H2AJ |
| HIST1H2AM |
| HIST1H2BC |
| HIST2H2AC |
| HLA-DRB5 |
| HLA-E |
| HLA-F |
| HMGB2 |
| HOMER3 |
| HOXB7 |
| HSBP1 |
| HSDL2 |
| HSPA1A |
| HSPB1 |
| HTATIP2 |
| ID2 |
| ID3 |
| IFITM4P |
| IGF2R |
| IGHA1 |
| IGHD |
| IGHM |
| IL13RA1 |
| IL18R1 |
| IL1R2 |
| IL23A |
| IL7R |
| IMPA2 |
| IMPDH1 |
| INCA |
| IRAK3 |
| ISG20 |
| ITM2C |
| JDP2 |
| KCNE3 |
| KCNG1 |
| KCNJ15 |
| KIAA0319L |
| KIAA1430 |
| KIAA1833 |
| KLF6 |
| KLHL3 |
| KLRC4 |
| KSR1 |
| LAG3 |
| LAMP2 |
| LAT2 |
| LCK |
| LHPP |
| LILRA2 |
| LILRB3 |
| LILRP2 |
| LIMS2 |
| LIN7A |
| LIN7B |
| LOC137886 |
| LOC149703 |
| LOC150166 |
| LOC153546 |
| LOC220433 |
| LOC389641 |
| LOC401233 |
| LOC401357 |
| LOC439949 |
| LOC440104 |
| LOC440348 |
| LOC440731 |
| LOC497190 |
| LOC644246 |
| LOXL2 |
| LPGAT1 |
| LRRK2 |
| LSM10 |
| LSM7 |
| LST1 |
| LTBP2 |
| LTBP3 |
| LY96 |
| MACF1 |
| MAGED1 |
| MAGED2 |
| MAGEH1 |
| MAK |
| MAN1C1 |
| MAN2A2 |
| MAP1LC3B |
| MAP3K2 |
| MAP3K3 |
| MAP4K4 |
| MAPK14 |
| MAPK8IP1 |
| 7-Mar |
| 9-Mar |
| MAX |
| MBOAT2 |
| MCL1 |
| MEA1 |
| MEGF10 |
| METTL9 |
| MGAM |
| MGC14425 |
| MLKL |
| MLSTD2 |
| MMD |
| MME |
| MMP9 |
| MNDA |
| MORC3 |
| MOSC1 |
| MOSPD2 |
| MPZL1 |
| MRLC2 |
| MRPL42P5 |
| MRPL53 |
| MSRB2 |
| MST150 |
| MUC20 |
| MUM1 |
| MXD1 |
| MYBPH |
| MYC |
| MYH14 |
| MYL6 |
| MYO15B |
| MYO1F |
| MYO1G |
| NAPSA |
| NAPSB |
| NBPF11 |
| NCF4 |
| NDRG2 |
| NDUFB3 |
| NDUFS8 |
| NFATC1 |
| NFIL3 |
| NGFRAP1 |
| NIN |
| NMI |
| NMT2 |
| NOVA1 |
| NPIP |
| NRBF2 |
| NRIP3 |
| NRP1 |
| NRSN2 |
| NUDT16 |
| OLIG1 |
| OR4C15 |
| OR52B2 |
| OSBPL2 |
| OSBPL6 |
| OSTF1 |
| OXNAD1 |
| PACSIN2 |
| PADI4 |
| PARP1 |
| PDCD7 |
| PDE9A |
| PDK2 |
| PDLIM7 |
| PELI1 |
| PFDN5 |
| PFKFB3 |
| PGD |
| PHB |
| PHC2 |
| PHF5A |
| PHGDH |
| PIK3C2B |
| PIM2 |
| PISD |
| PITPNA |
| PLA2G4A |
| PLA2G7 |
| PLAG1 |
| PLD3 |
| PLEKHA1 |
| PLEKHM1 |
| PLXNC1 |
| POLR2A |
| PPP1R12B |
| PPP4R2 |
| PRAP1 |
| PRKAR1A |
| PRKAR1B |
| PRKCA |
| PRKCD |
| PRKDC |
| PRKY |
| PRSS23 |
| PSMB9 |
| PSMD8 |
| PTEN |
| PTOV1 |
| PTPRCAP |
| PTPRK |
| PTPRM |
| PXK |
| PYCARD |
| PYGL |
| QPCT |
| QPRT |
| RAB24 |
| RAB27A |
| RAB31 |
| RAB32 |
| RABGAP1L |
| RABIF |
| RAC1 |
| RAC2 |
| RAI1 |
| RALB |
| RALGDS |
| RARA |
| RASSF2 |
| RBP7 |
| RCC2 |
| REEP5 |
| REPS2 |
| RFWD2 |
| RGS16 |
| RGS2 |
| RHOG |
| RHOH |
| RIMS4 |
| RIT1 |
| RMND5A |
| RNF130 |
| RNF182 |
| RNF24 |
| ROCK2 |
| ROPN1L |
| RPL17 |
| RPL18A |
| RPL22 |
| RPL31 |
| RPL34 |
| RPL36A |
| RPL37 |
| RPL39 |
| RPS10 |
| RPS15 |
| RPS21 |
| RPS27 |
| RPS27A |
| RPS28 |
| RPS4X |
| RPUSD2 |
| RRN3 |
| RTN3 |
| S100A11 |
| S100A12 |
| S100A8 |
| S100A9 |
| S100P |
| SAMSN1 |
| SAP30 |
| SCRN2 |
| SDCBP |
| SEC14L1 |
| SEC22B |
| SEPX1 |
| SERINC1 |
| SERPINB1 |
| SERPINB8 |
| SERPINE1 |
| SF3B14 |
| SFT2D1 |
| SGCE |
| SH2D5 |
| SLA |
| SLC16A3 |
| SLC1A7 |
| SLC22A15 |
| SLC22A4 |
| SLC25A37 |
| SLC2A10 |
| SLC2A14 |
| SLC2A8 |
| SLC35B4 |
| SLC37A3 |
| SLC40A1 |
| SLC45A2 |
| SLC8A1 |
| SLIT3 |
| SMARCD3 |
| SMC1A |
| SMUG1 |
| SOD2 |
| SP100 |
| SPIB |
| SPRR2C |
| SRM |
| SRPK1 |
| SSBP4 |
| ST6GAL1 |
| STAT5A |
| STC1 |
| STK17B |
| STMN1 |
| STX10 |
| STX3 |
| SULT1B1 |
| SYNCRIP |
| SYT15 |
| TAF9B |
| TALDO1 |
| TANK |
| TARP |
| TAX1BP1 |
| TBCD |
| TBL1XR1 |
| TCEAL1 |
| TCF3 |
| TCF7 |
| THBD |
| TLR2 |
| TLR8 |
| TM7SF2 |
| TMEM102 |
| TMEM48 |
| TMEM49 |
| TMEM68 |
| TMEM86A |
| TNFAIP6 |
| TNFRSF10A |
| TP53I11 |
| TP53TG3 |
| TPST1 |
| TRA@ |
| TRAPPC2L |
| TREM1 |
| TRIB1 |
| TRIM7 |
| TSEN34 |
| TSPAN13 |
| TSPAN16 |
| TSPAN33 |
| TUFM |
| TXN |
| TYROBP |
| U2AF1 |
| UBC |
| UBE2D3 |
| UBE2G2 |
| UBL5 |
| UBQLN1 |
| UCP2 |
| UPF3A |
| URG4 |
| USP11 |
| USP53 |
| USP6 |
| VKORC1 |
| VWCE |
| WDFY3 |
| WDR18 |
| XKR8 |
| XPR1 |
| YOD1 |
| YPEL4 |
| ZBED1 |
| ZCCHC6 |
| ZNF135 |
| ZNF234 |
| ZNF346 |
| ZNF438 |
| ZNF550 |
| ZNF618 |

Table S4. Results of Gene Ontology Analysis of 655 Microarray Derived Genes

| Accession | Term | p-value | corr p-value | Ontology |
| --- | --- | --- | --- | --- |
| 2376 | immune system process | 8.43E-10 | 1.45E-06 | BP |
| 6455 | translational elongation | 5.55E-09 | 4.78E-06 | BP |
| 43067 | regulation of programmed cell death | 1.44E-06 | 6.49E-04 | BP |
| 6955 | immune response | 1.51E-06 | 6.49E-04 | BP |
| 42981 | regulation of apoptosis | 2.65E-06 | 9.13E-04 | BP |
| 2521 | leukocyte differentiation | 5.56E-06 | 1.59E-03 | BP |
| 6690 | icosanoid metabolic process | 6.44E-06 | 1.59E-03 | BP |
| 46649 | lymphocyte activation | 1.25E-05 | 2.61E-03 | BP |
| 50793 | regulation of developmental process | 1.37E-05 | 2.61E-03 | BP |
| 46650 | lymphocyte differentiation | 2.10E-05 | 3.57E-03 | BP |
| 2682 | regulation of immune system process | 2.28E-05 | 3.57E-03 | BP |
| 2684 | positive regulation of immune system process | 2.85E-05 | 3.77E-03 | BP |
| 45321 | leukocyte activation | 2.85E-05 | 3.77E-03 | BP |
| 2694 | regulation of leukocyte activation | 5.96E-05 | 7.33E-03 | BP |
| 6952 | defense response | 6.87E-05 | 7.55E-03 | BP |
| 44260 | cellular macromolecule metabolic process | 7.65E-05 | 7.55E-03 | BP |
| 50865 | regulation of cell activation | 8.16E-05 | 7.55E-03 | BP |
| 43449 | alkene metabolic process | 8.33E-05 | 7.55E-03 | BP |
| 6691 | leukotriene metabolic process | 8.33E-05 | 7.55E-03 | BP |
| 43066 | negative regulation of apoptosis | 9.71E-05 | 8.36E-03 | BP |
| 43068 | positive regulation of programmed cell death | 1.13E-04 | 8.77E-03 | BP |
| 43069 | negative regulation of programmed cell death | 1.17E-04 | 8.77E-03 | BP |
| 51249 | regulation of lymphocyte activation | 1.17E-04 | 8.77E-03 | BP |
| 7243 | protein kinase cascade | 1.34E-04 | 9.62E-03 | BP |
| 30031 | cell projection biogenesis | 1.69E-04 | 1.12E-02 | BP |
| 7242 | intracellular signaling cascade | 1.69E-04 | 1.12E-02 | BP |
| 2245 | response to wounding | 2.15E-04 | 1.37E-02 | BP |
| 31347 | regulation of defense response | 2.27E-04 | 1.40E-02 | BP |
| 43065 | positive regulation of apoptosis | 2.66E-04 | 1.55E-02 | BP |
| 44267 | cellular protein metabolic process | 2.78E-04 | 1.55E-02 | BP |
| 6954 | inflammatory response | 2.78E-04 | 1.55E-02 | BP |
| 6917 | induction of apoptosis | 3.12E-04 | 1.63E-02 | BP |
| 1775 | cell activation | 3.24E-04 | 1.63E-02 | BP |
| 31349 | positive regulation of defense response | 3.31E-04 | 1.63E-02 | BP |
| 12502 | induction of programmed cell death | 3.31E-04 | 1.63E-02 | BP |
| 9605 | response to external stimulus | 3.92E-04 | 1.88E-02 | BP |
| 51094 | positive regulation of developmental process | 4.04E-04 | 1.88E-02 | BP |
| 6916 | anti-apoptosis | 4.35E-04 | 1.92E-02 | BP |
| 19538 | protein metabolic process | 4.35E-04 | 1.92E-02 | BP |
| 42110 | T cell activation | 4.45E-04 | 1.92E-02 | BP |
| 48519 | negative regulation of biological process | 5.88E-04 | 2.47E-02 | BP |
| 30097 | hemopoiesis | 6.21E-04 | 2.55E-02 | BP |
| 6950 | response to stress | 6.97E-04 | 2.79E-02 | BP |
| 2520 | immune system development | 7.18E-04 | 2.81E-02 | BP |
| 2696 | positive regulation of leukocyte activation | 8.09E-04 | 3.03E-02 | BP |
| 50867 | positive regulation of cell activation | 8.09E-04 | 3.03E-02 | BP |
| 51869 | response to stimulus | 8.93E-04 | 3.27E-02 | BP |
| 30183 | B cell differentiation | 1.02E-03 | 3.65E-02 | BP |
| 48583 | regulation of response to stimulus | 1.07E-03 | 3.75E-02 | BP |
| 45082 | positive regulation of interleukin-10 biosynthetic process | 1.11E-03 | 3.75E-02 | BP |
| 45074 | regulation of interleukin-10 biosynthetic process | 1.11E-03 | 3.75E-02 | BP |
| 42035 | regulation of cytokine biosynthetic process | 1.25E-03 | 4.05E-02 | BP |
| 50776 | regulation of immune response | 1.25E-03 | 4.05E-02 | BP |
| 43412 | biopolymer modification | 1.44E-03 | 4.60E-02 | BP |
| 5515 | protein binding | 4.00E-07 | 2.87E-04 | MF |
| 4197 | cysteine-type endopeptidase activity | 2.68E-05 | 9.63E-03 | MF |
| 30693 | caspase activity | 4.51E-05 | 1.08E-02 | MF |
| 5829 | cytosol | 6.01E-09 | 1.97E-06 | CC |
| 5830 | cytosolic ribosome | 2.16E-07 | 3.54E-05 | CC |
| 44445 | cytosolic part | 2.18E-06 | 2.15E-04 | CC |
| 5737 | cytoplasm | 2.63E-06 | 2.15E-04 | CC |
| 30873 | cytosolic small ribosomal subunit | 4.76E-05 | 3.11E-03 | CC |
| 33279 | ribosomal subunit | 6.09E-05 | 3.32E-03 | CC |
| 44444 | cytoplasmic part | 3.24E-04 | 1.51E-02 | CC |
| 16461 | unconventional myosin complex | 5.65E-04 | 2.31E-02 | CC |
| 30498 | cytosolic large ribosomal subunit | 7.93E-04 | 2.88E-02 | CC |
| 44424 | intracellular part | 1.47E-03 | 4.79E-02 | CC |
